# A Retrospective Cohort Study on the Clinical Course of Patients With Moderate-Type COVID-19

**DOI:** 10.3389/fpubh.2021.593109

**Published:** 2021-04-26

**Authors:** Xiaohua Liao, Xin Lv, Cheng Song, Mao Jiang, Ronglin He, Yuanyuan Han, Mengyu Li, Yan Zhang, Yupeng Jiang, Jie Meng

**Affiliations:** ^1^Department of Pulmonary and Critical Care Medicine, The Third Xiangya Hospital of Central South University, Changsha, China; ^2^Department of Pulmonary and Critical Care Medicine, Central Hospital of Wuhan City, Wuhan, China; ^3^Department of Nephrology, Xiangya Hospital of Central South University, Changsha, China; ^4^Organ Fibrosis Key Laboratory of Hunan Province, Changsha, China

**Keywords:** COVID-19, SARS-CoV-2, moderate type, treatment, clinical course

## Abstract

**Background:** A large number of people contracted moderate-type COVID-19 around the world. However, to our knowledge no studies have covered the clinical course of patients with moderate-type COVID-19. This study describes the clinical course of moderate-type patients with COVID-19 from Wuhan City and Yiyang City, and explores factors relevant to the length of hospitalization and symptoms relief.

**Methods:** The study analyzed the clinical course of 107 moderate-type patients with COVID-19 from the outbreak area (Wuhan) and the imported area (Yiyang), and used automatic linear modeling and multivariate linear regression analysis to explore the factors relevant to the length of hospitalization and symptoms relief. Furthermore, we created a scoring system to value the length of hospitalization and symptoms relief.

**Results:** Lymphopenia, elevated C-reactive protein, increased LDH, bilateral lung GGO (ground glass opacity), and lung consolidation were more likely to appear in ordinary inpatients with moderate-type COVID-19 from Wuhan (*P* < 0.05), compared to infected medical staff from Wuhan and ordinary inpatients with moderate-type COVID-19 from Yiyang. Meanwhile, the length of hospitalization and symptoms relief was longer in ordinary patients with moderate-type COVID-19 from Wuhan (*P* < 0.05). Onset of symptoms to admission, ESR, leucocytes count, and bilateral lung GGO were linearly related to the length of hospitalization (*P* < 0.05); onset of symptoms to admission, leucocytes count, bilateral lung GGO, and lung consolidation were linearly related to the length of symptoms relief (*P* < 0.05). By using the scoring system, we found that the time of hospitalization and symptoms relief lengthened as the scores increased.

**Conclusions:** This study described the clinical course of patients with moderate-type COVID-19, and found that ordinary patients with moderate-type COVID-19 in outbreak areas were more serious and needed stronger treatment and longer treatment time. Onset of symptoms to admission, ESR, leucocytes count, and bilateral lung GGO can be effective predictors of the length of hospitalization. And onset of symptoms to admission, leucocytes count, bilateral lung GGO, and lung consolidation can be effective predictors of the amount of time until symptoms relief. Most importantly, we have created a scoring system, which could contribute to the diagnosis and treatment of COVID-19.

## Introduction

In December 2019, an emerging pneumonia caused by severe acute respiratory syndrome-novel coronavirus 2 (SARS-CoV-2) infection, named coronavirus disease-19 (COVID-19), appeared in Wuhan, Hubei Province (1). Most of the early patients were exposed to the Huanan seafood wholesale market (2). Like MERS-COV and SARS-COV, the coronavirus (SARS-CoV2) belonged to the beta coronavirus family (3). Current research suggested that it may have originated from bats and pangolins (4, 5). SARS-CoV-2 can be passed from person to person (6). The World Health Organization has identified COVID-19 as a global pandemic (7). So far, around 100,000 cases of COVID-19 have been reported in China, and around 119,000,000 cases have been reported outside of China, which posed an enormous threat to human health.

The clinical features of COVID-19 have been reported in severe and non-severe patients, as well as in surviving and deceased patients (8, 9). However, at present no studies have reported the clinical course of patients with moderate-type COVID-19. According to the 7th Chinese edition of the COVID-19 Diagnosis and Treatment Guidelines, patients are divided into four types based on their results of clinical examination, including mild-type, moderate-type, serious-type, and critical-type (10). There was a large number of patients with moderate-type COVID-19 around the world. A study reported that 262 patients were confirmed to have COVID-19 from 57 hospitals in Beijing, and 192 (73%) of them had moderate-type COVID-19 (11). In another study about COVID-19 from Renmin Hospital of Wuhan University, moderate patients also accounted for 56% (12). Our data showed that 65% (66/101) of patients in Central Hospital of Wuhan and 72% (43/60) of patients in Yiyang had moderate-type COVID-19. Studies from Egypt, USA, and Spain reported that 51.5, 36, and 44.6% of patients had moderate cases, respectively (13–15).

It is meaningful to pay attention to patients with moderate-type COVID-19. On the one hand, the large number of patients with moderate-type COVID-19 is a dangerous source of infection. On the other hand, the sooner the moderate-type patients are cured, the sooner they can be released from isolation and return to normal life, which is beneficial to reduce social panic and promote the restoration of social and economic life. For patients with moderate-type COVID-19, the relevant factors for disease progression have not been completely understood, which led to the vagueness of criteria for these patients to hospital admission in the US CDC (Centers for Disease Control and Prevention) COVID-19 guidelines (16). In China, almost all patients with COVID-19 received free treatment in the hospital, which is beneficial for us to describe and compare the clinical course of patients with moderate-type COVID-19 in the outbreak area (Wuhan) and in the imported area (Yiyang).

The aims of the study were to describe the clinical course of moderate-type patients with COVID-19 from Wuhan City and Yiyang City, and to explore factors relevant to the length of hospitalization and symptoms relief. The results of our research can be helpful for the appropriate treatment of moderate-type patients with COVID-19 from outbreak areas and imported areas. The study also found related factors to predict the length of hospitalization and symptoms relief, and offered an effective scoring system to value the severity and treatment of COVID-19.

## Methods

### Study Design and Participants

A total of 107 moderate-type patients discharged from December 16, 2019 to February 21, 2020 were enrolled in this study. This retrospective cohort study included three groups of moderate-type patients with COVID-19: ordinary inpatients from the Central Hospital of Wuhan, infected medical staff from the Central Hospital of Wuhan, and ordinary inpatients from Yiyang City. Due to death after pneumonia exacerbation, two cases of ordinary inpatients with moderate-type COVID-19 from Wuhan were excluded from the study. Among all the confirmed patients in Yiyang, 43 cases of inpatients with moderate-type COVID-19 were enrolled, and none of them were medical workers. At present, all the patients with COVID-19 in Yiyang have been completely cured and discharged. All data were collected through the hospital electrical records system. This study was approved by the Ethic Committee of COVID-19 designated hospitals (Ethic number: K-20040).

### Data Collection

Demographic data, medical history, contact history, comorbidities, symptoms, signs, laboratory tests (such as leucocytes count, lymphocytes count, C-reactive protein, erythrocyte sedimentation rate (ESR), D-dimer, lactate dehydrogenase (LDH), creatine kinase (CK), aspartate aminotransferase (AST), alanine aminotransferase (ALT), etc.), chest computed tomography (CT) scans, and hospitalization treatment (i.e., antiviral treatment, glucocorticoid therapy, respiratory support, intravenous immunoglobulin therapy, and antibiotic therapy) were collected from the electronic medical records system. Unilateral GGO (ground glass opacity), bilateral lung GGO, and lung consolidation were all based on the results from radiologists. All data were checked by two physicians (XLV and ML).

### Definition

All the enrolled patients were diagnosed and discharged based on the 7th Chinese edition of the COVID-19 Diagnosis and Treatment Guidelines (10). The diagnostic criteria of moderate-type COVID-19 included: presenting clinical symptoms of the respiratory tract; having radiological evidence of viral pneumonia; respiratory rate <30 per min; oxygen saturation >93% when breathing ambient air, or ratio of arterial oxygen tension (PaO_2_) over inspiratory oxygen fraction (FIO_2_) >300 mmHg (1 mmHg equals to 0.133 kPa); lung imaging indicating multilocular lesions or progression of lesions <50% within 48 h. The criteria for discharge included: at least 3 days without fever, substantial improvement in both lungs in chest CT, clinical remission of respiratory symptoms, and two pharyngeal swab samples obtained at least 24 h apart that were negative for SARS-CoV-2 RNA (10). The date of onset was defined as the day when symptoms appeared. Onset of symptoms to admission was defined as the time from the day of symptoms onset to the day when patients were admitted to the hospital. The length of symptoms relief was defined as the time from the day of symptoms onset to the day when symptoms were improved to discharge.

### Laboratory Procedures

To confirm SARS-CoV-2 infection, pharyngeal swab specimens provided by Wuhan Central Hospital or designated Hospitals of Yiyang were detected by RT-PCR in the local Center for Disease Control and Prevention. During hospitalization, the doctor re-examined the patients based on the alleviation of symptoms and chest CT scans. The frequency of laboratory tests and imaging tests was determined by the attending physicians.

### Statistical Analysis

Continuous and categorical variables were presented as median (IQR) and *n* (%), respectively. We used the Whitney U test, χ^2^ test, or Fisher's exact test to compare the differences of inpatients with moderate-type COVID-19 from Wuhan City and Yiyang City. To explore factors relevant to the length of hospitalization and symptoms relief, we used automatic linear modeling. Then the predicted factors were subjected to multiple linear regression analysis. The correlation scores of these factors was decided by their regression coefficient (β). When the regression coefficient (β) was < 0.05, the correlation score was 1 point; when the regression coefficient (β) was < 0.01, the correlation score was 2 points. The total correlation score was calculated for each patient by summation of the score points. And comparisons between the total score groups were analyzed with one-way ANOVA, and comparisons between two groups were analyzed with a least significant difference (LSD) test. All statistics were analyzed using SPSS (the version 20.0 software). For unadjusted comparisons, a two-sided α of < 0.05 was considered statistically significant.

## Results

### Comparison of the Characteristics of Moderate-Type COVID-19 in Ordinary Patients and Infected Medical Staff From Wuhan

Ordinary hospitalized patients with moderate-type COVID-19 were older than infected medical staff [51 years (IQR, 41.5–59.75) vs. 34 years (IQR, 28–37)] (*P* < 0.001), and there was no significant difference in gender distribution (Table 1). Six ordinary patients had visited the Huanan seafood wholesale market, and none of infected medical staff had been to the seafood market. Ordinary patients had more comorbidities [5 (17.86%) vs. 0] (*P* = 0.030), as well as a longer time from the onset of symptoms to hospital admission [6 days (IQR, 3.25–8.75) vs. 3 days (IQR, 2-5.75)] (*P* = 0.027) (Table 1). Compared with ordinary patients, infected medical staff were more prone to suffer from fatigue [4 (14.29%) vs. 14 (38.89%)] (*P* = 0.030) and myalgia [2 (7.14%) vs. 16 (44.44%)] (*P* = 0.001). Other symptoms presented no significant differences between the two groups (Table 1). Compared with infected medical staff, the levels of AST, C-reactive protein, ESR, and LDH in ordinary patients were significantly increased (*P* < 0.01), and lymphocytes were significantly decreased (*P* < 0.001) (Table 2). Chest CT scans showed that ordinary patients had more bilateral lung GGO and lung consolidation (*P* < 0.01), while unilateral GGO was more likely to appear in infected medical staff (*P* < 0.01) (Table 2). There was no significant differences between treatment (i.e., antiviral treatment, glucocorticoid therapy, respiratory support, intravenous immunoglobulin therapy, and antibiotic therapy) (Table 1). However, compared with infected medical staff, the length of hospitalization of ordinary patients was longer [26 days (IQR, 17.75–30.25) vs. 21 days (IQR, 18–25)] (*P* = 0.041), as well as the length of symptoms relief [32 days (IQR, 24.5–36) vs. 26 days (IQR, 23–28)] (*P* = 0.002) (Table 1).

**Table 1:** Demographic, clinical characteristics, treatment, and outcome of inpatients with moderate-type COVID-19 from Wuhan.

	**28 Wuhan ordinary patients**	**36 Wuhan medical staff**	***P*-value**
Onset of symptoms to admission, median(IQR), d	6 (3.25–8.75), range from 1 to 17	3 (2–5.75), range from 1 to 14	0.027
Age, median (IQR), years	51 (41.5–59.75),range from 20 to 73	34 (28–37),range from 22 to 47	0.001
**Sex**			
Male	11 (39.29%)	16 (44.44%)	0.678
Female	17 (60.71%)	20 (55.56%)	
Huanan Seafood Wholesale Market exposure	6 (21.43%)	0	0.013
**Signs and symptoms**			
Fever	21 (75%)	27 (75.00%)	1
Cough	16 (57.14%)	23 (63.89%)	0.583
Sputum	3 (10.71%)	2 (5.56%)	0.769
Chest distress	3 (10.71%)	5 (13.89%)	1
Fatigue	4 (14.29%)	14 (38.89%)	0.03
Myalgia	2 (7.14%)	16 (44.44%)	0.001
Headache	1 (3.57%)	7 (19.44%)	0.128
Diarrhea	2 (7.14%)	2 (5.56%)	1
Dizziness	1 (3.57%)	0	0.438
Anorexia	1 (3.57%)	0	0.438
Rhinobyon	0	3 (8.33%)	0.333
Comorbidities	5 (17.86%)	0	0.030
Hypertension	3 (10.71%)	0	0.333
Cerebrovascular disease	1 (3.57%)	0	0.438
Malignancy	1 (3.57%)	0	0.438
**Treatment**			
Normal flow of oxygen	27 (96.43%)	30 (83.33%)	0.207
Glucocorticoids	26 (92.86%)	28 (77.78%)	0.193
Intravenous immunoglobulin therapy	15 (53.57%)	24 (66.67%)	0.287
Antibiotic treatment	28 (100%)	36 (100%)	
Antiviral treatment	28 (100%)	36 (100%)	
**Clinical outcome**			
Discharged	28 (100%)	36 (100%)	
The length of hospitalization (IQR), d	26 (17.75–30.25), range from 12 to 36	21 (18-25), range from 6 to 31	0.041
The length of symptoms relief (IQR), d	32 (24.5–36), range from 17 to 42	26 (23-28), range from 14 to 36	0.002

**Table 2:** Laboratory examination of inpatients with moderate-type COVID-19 from Wuhan.

	**28 Wuhan**	**36 Wuhan**	***P*-value**
	**ordinary patients**	**medical staff**	
**Leucocytes count (×10^9^/L; normal range 3.5~9.5) (IQR)**	4.06 (3.18–4.99)	4.78 (3.27–5.89)	0.250
Increased	1 (3.57%)	0	0.438
Decreased	9 (32.14%)	10 (27.78%)	0.705
**Lymphocytes count (×10^9^/L; normal range 1.1~3.2) (IQR)**	0.71 (0.59–0.98)	1.29 (0.91–1.45)	0.001
Decreased	22 (78.57%)	13 (36.11%)	0.001
**D-dimer (mg/L; normal range 0~0.5) (IQR)**	0.32 (0.19–0.83)	0.25 (0.17–0.45)	0.149
Increased	9 (32.14%)	8 (22.22%)	0.373
**Albumin (g/L; normal range 40~55)(IQR)**	40.15 (37.45–43.05)	41.65 (39.25–44.98)	0.054
Decreased	13 (46.43%)	10 (27.78%)	0.123
**Alanine aminotransferase (U/L; normal range 9~50) (IQR)**	21.35 (14.73–37.60)	17.6 (11.03–23.25)	0.142
Increased	6 (21.43%)	4 (11.11%)	0.435
Decreased	1 (3.57%)	3 (8.33%)	0.795
**Aspartate aminotransferase (U/L; normal range 15~40) (IQR)**	26.75 (18.95–41.35)	17.5 (14.63–22.83)	0.001
Increased	7 (25.00%)	3 (8.33%)	0.140
Decreased	2 (7.14%)	10 (27.78%)	0.036
**C-reactive protein (mg/dL; normal range 0~0.6) (IQR)**	1.12 (0.2–2.80)	0.03 (0.01–0.11)	0.001
Increased	15 (53.57%)	0	0.001
**Erythrocyte sedimentation rate (mm/h; normal range 0~15) (IQR)**	37 (13.5–54)	10 (9–13.5)	0.001
Increased	20 (71.43%)	1 (2.78%)	0.001
**Creatine kinase (U/L; normal range 0~171) (IQR)**	91 (46–134)	72.30 (42.50–95.65)	0.253
Increased	3 (10.71%)	4 (11.11%)	1
**Creatine kinase-MB (U/L; normal range 0~25) (IQR)**	8.5 (6.00–10.00)	7.70 (4.50–9.45)	0.260
**Lactate dehydrogenase (U/L; normal range 120~250) (IQR)**	219 (188–242)	141.00 (120.00–170.50)	0.001
Increased	6 (21.43%)	2 (5.56%)	0.128
Decreased	0	7 (19.44%)	0.039
**Chest CT scans**			
Bilateral lung GGO	27 (96.43%)	23 (63.89%)	0.002
Single lung GGO	1 (3.57%)	11 (30.56%)	0.006
Pulmonary consolidation	20 (71.43%)	0	0.001

### Comparison of the Characteristics of Moderate-Type COVID-19 in Ordinary Patients From Wuhan and Yiyang

There was no significant difference in ordinary patients from Wuhan (28 cases) and Yiyang (43 cases) in age, gender, comorbidities, and the time from the onset of symptoms to hospital admission (Table 3). The ordinary patients from Wuhan were more prone to present with fever (*P* = 0.001), and other symptoms had no significant differences between the two groups (Table 3). Compared with ordinary patients from Yiyang, ordinary patients from Wuhan had fewer white blood cells and lymphocytes (*P* < 0.01), while C-reactive protein and LDH were increased significantly (*P* < 0.01) (Table 4). Chest CT scans showed that more bilateral lung GGO and lung consolidation were seen in Wuhan ordinary patients (*P* < 0.01), while unilateral GGO was more likely to appear in Yiyang patients (*P* < 0.01) (Table 4). All patients in both groups received antiviral treatment, and the number of patients receiving a normal flow of oxygen, antibiotics, systemic glucocorticoid, and intravenous immunoglobulin was more in Wuhan than that in Yiyang (*P* < 0.001) (Table 3). Compared with Yiyang patients, the length of hospitalization of Wuhan ordinary patients was longer {26 days (IQR, 17.75–30.25) vs. 11 days [IQR, (8–15)]} (*P* < 0.001), as well as the length of symptoms relief [32 days (IQR, 24.5–36) vs. 16 days (IQR, 14–20)] (*P* < 0.001) (Table 3).

**Table 3:** Demographic, clinical characteristics, treatment, and outcome of ordinary patients with moderate-type COVID-19 from Wuhan and Yiyang.

	**28 Wuhan ordinary patients**	**43 Yiyang ordinary patients**	***P*-value**
Onset of symptoms to admission, median (IQR), d	6 (3.25–8.75), range from 1 to 17	6 (3-8), range from 0 to 29	0.657
Age, median (IQR), years	51 (41.5–59.75),range from 20 to 73	46 (37–55),range from 15 to 80	0.182
**Sex**			
Male	11 (39.29%)	17 (39.5%)	0.983
Female	17 (60.71%)	26 (60.5%)	
History of seafood market contact	6 (21.43%)	0	0.006
**Signs and symptoms**			
Fever	21 (75%)	15 (35.9%)	0.001
Cough	16 (57.14%)	25 (58.1%)	0.934
Sputum	3 (10.71%)	13 (30.2%)	0.054
Dyspnea	0	1 (2.3%)	1
Chest distress	3 (10.71%)	0	0.112
Fatigue	4 (14.29%)	10 (23.2%)	0.353
Myalgia	2 (7.14%)	5 (11.6%)	0.832
Headache	1 (3.57%)	3 (7%)	0.935
Diarrhea	2 (7.14%)	2 (4.7%)	1
Dizziness	1 (3.57%)	4 (9.3%)	0.654
Anorexia	1 (3.57%)	0	0.394
No symptoms	0	7 (16.3%)	0.066
**Comorbidities**			
Coronary atherosclerosis	0	3 (7%)	0.410
Hypertension	3 (10.71%)	4 (9.3%)	1
Cerebrovascular disease	1 (3.57%)	0	0.394
Diabetes	0	3 (7%)	0.112
Malignancy	1 (3.57%)	0	0.394
**Treatment**			
Normal flow of oxygen	27 (96.43%)	3 (7%)	0.001
Glucocorticoids	26 (92.86%)	9 (20.9%)	0.001
Intravenous immunoglobulin therapy	15 (53.57%)	5 (11.6%)	0.001
Antibiotic treatment	28 (100%)	17 (39.5%)	0.001
Antiviral treatment	28 (100%)	43 (100%)	
**Clinical outcome**			
Discharged	28 (100%)	43 (100%)	
The length of hospitalization (IQR), d	26 (17.75–30.25), range from 12 to 36	11 (8-15), range from 4 to 20	0.001
The length of symptoms relief (IQR), d	32 (24.5–36), range from 17 to 42	16 (14-20) range from 9 to 39	0.001

**Table 4:** Laboratory examination of ordinary patients with moderate-type COVID-19 from Wuhan and Yiyang.

	**28 Wuhan ordinary patients**	**43 Yiyang ordinary patients**	***P*-value**
**Leucocytes count (×10^9^/L; normal range 3.5~9.5) (IQR)**	4.06 (3.18–4.99)	5.45 (4.06–7.51)	0.005
Increased	1 (3.57%)	4 (9.3%)	0.654
Decreased	9 (32.14%)	2 (4.7%)	0.005
**Lymphocytes count (×10^9^/L; normal range 1.1~3.2) (IQR)**	0.71 (0.59–0.98)	1.22 (0.96–1.53)	0.001
Decreased	22 (78.57%)	16 (37.2%)	0.001
**D-dimer (mg/L; normal range 0~0.5) (IQR)**	0.32 (0.19–0.83)	0.41 (0.19–0.68)	0.670
Increased	9 (32.14%)	12 (27.9%)	0.702
**Aspartate aminotransferase (U/L; normal range 15~40)(IQR)**	26.75 (18.95-41.35)	24 (19-34)	0.441
Increased	7 (25.00%)	4 (9.3%)	0.147
**C-reactive protein (mg/dL; normal range 0~0.6) (IQR)**	1.12 (0.2–2.80)	0.13 (0.05–0.97)	0.006
Increased	15 (53.57%)	12 (27.9%)	0.029
**Erythrocyte sedimentation rate (mm/h; normal range 0~15) (IQR)**	37 (13.5–54)	23 (13–44.1)	0.147
Increased	20 (71.43%)	30 (69.8%)	0.881
**Creatine kinase (U/L; normal range 0~171) (IQR)**	91 (46–134)	54.5 (37.25–84)	0.054
Increased	3 (10.71%)	5 (11.6%)	1
**Lactate dehydrogenase (U/L; normal range 120~250) (IQR)**	219 (188–242)	181 (155.5–223)	0.002
Increased	6 (21.43%)	3 (7.0%)	0.155
**Chest CT scans**			
Bilateral lung GGO	27 (96.43%)	10 (23.26%)	0.001
Single lung GGO	1 (3.57%)	14 (32.56%)	0.003
Pulmonary consolidation	20 (71.43%)	6 (13.95%)	0.001

Sixteen Yiyang patients had travel history in Wuhan. In order to verify whether the travel history affected the above results, we compared the characteristics of the patients. It was found that patients with travel history were more likely to present fatigue (*P* = 0.016). There were no obvious differences in other aspects (Supplementary Tables 1, 2).

### Analyzing the Relevant Factors of the Length of Hospitalization and Symptoms Relief of Inpatients With Moderate-Type COVID-19

**Figure 1:**
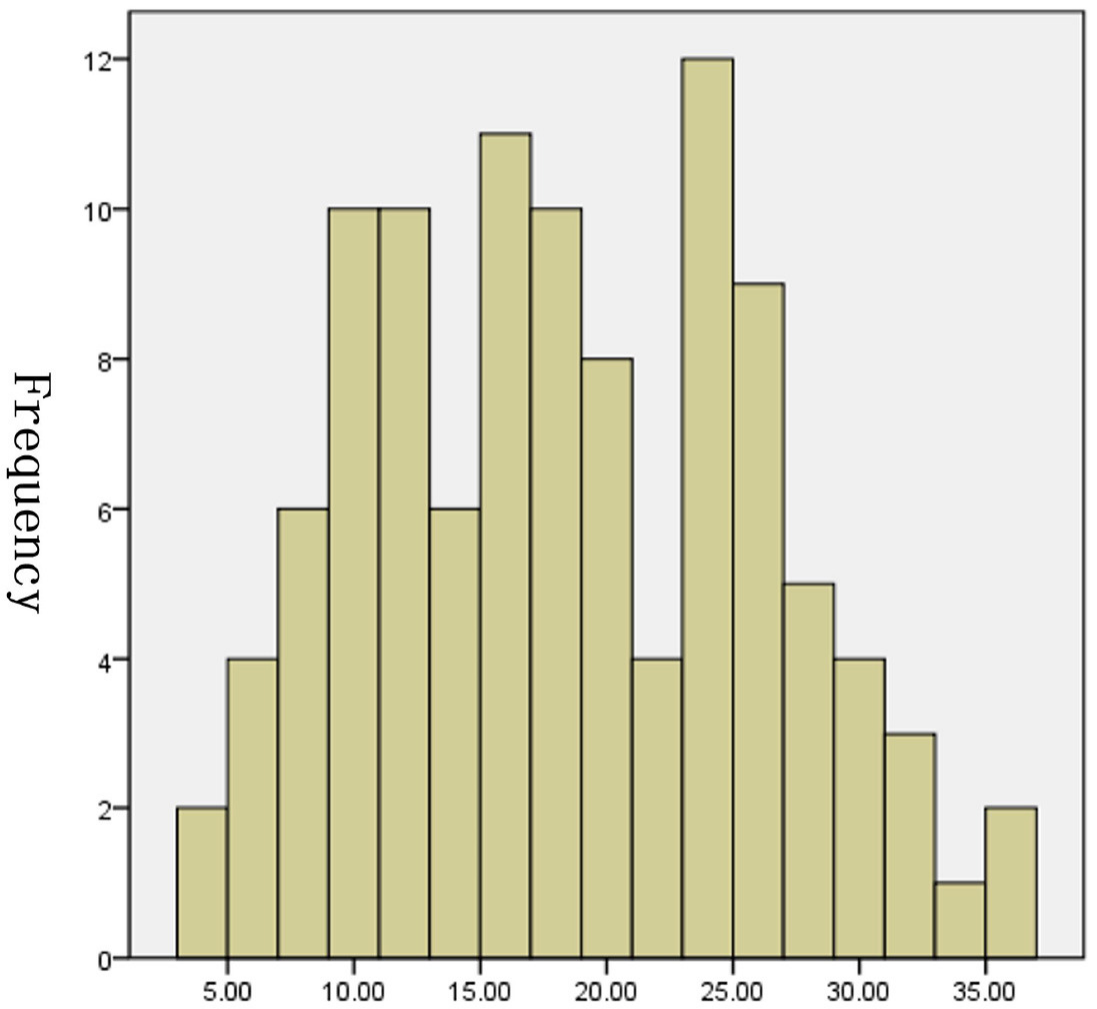
Histogram of the length of hospitalization (*P* = 0.035).

**Figure 2:**
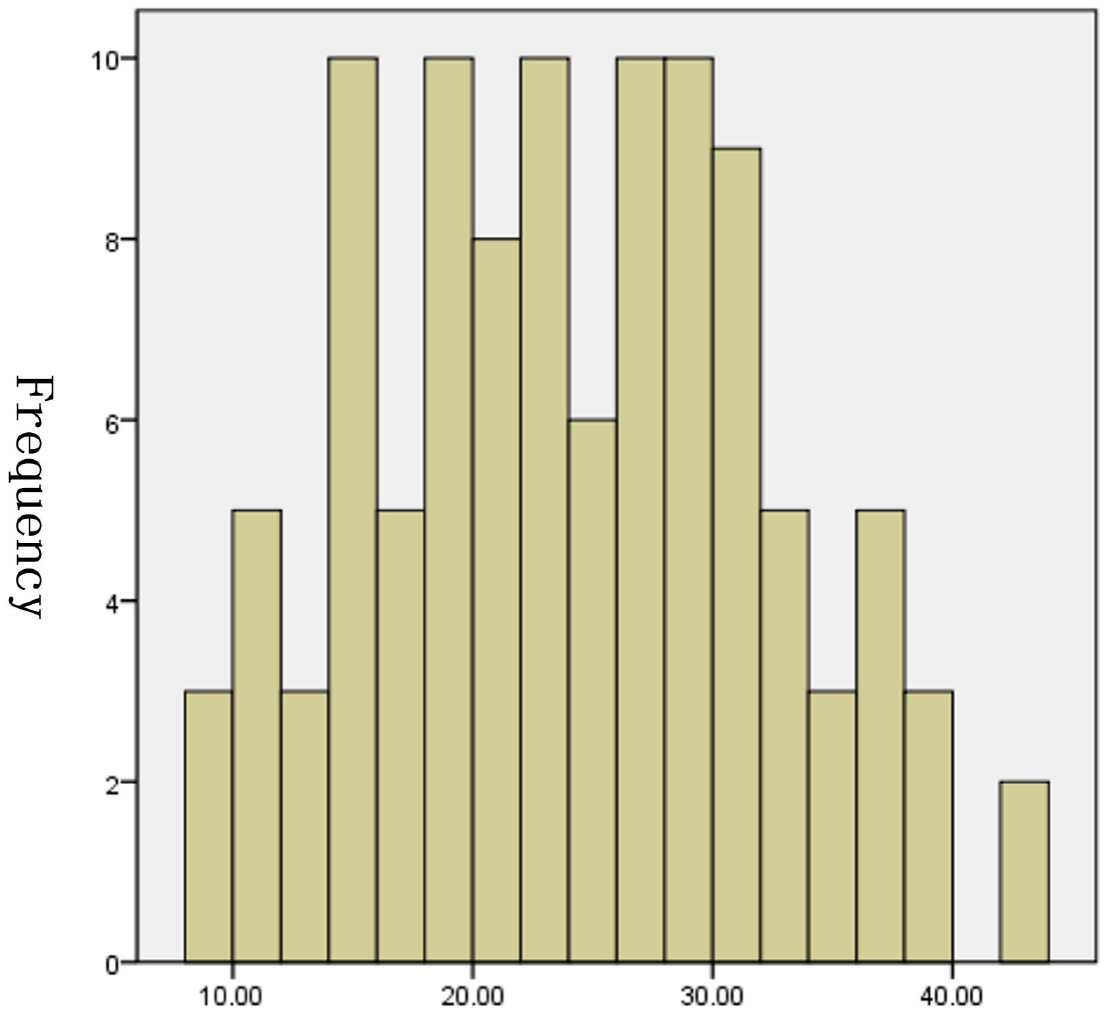
Histogram of the length of symptoms relief (*P* = 0.107).

**Figure 3:**
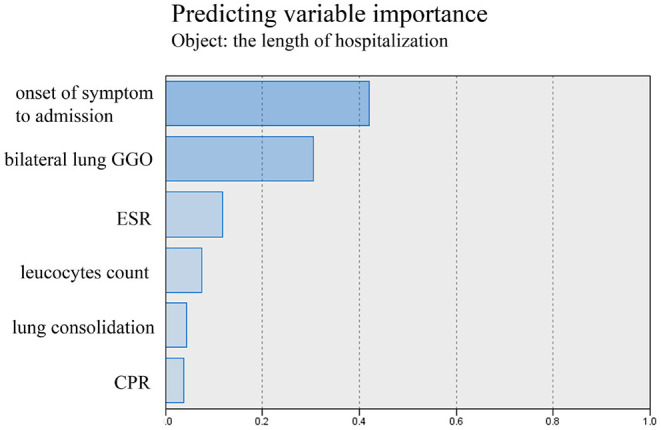
Using automatic linear modeling to predict relevant factors of the length of hospitalization.

**Figure 4:**
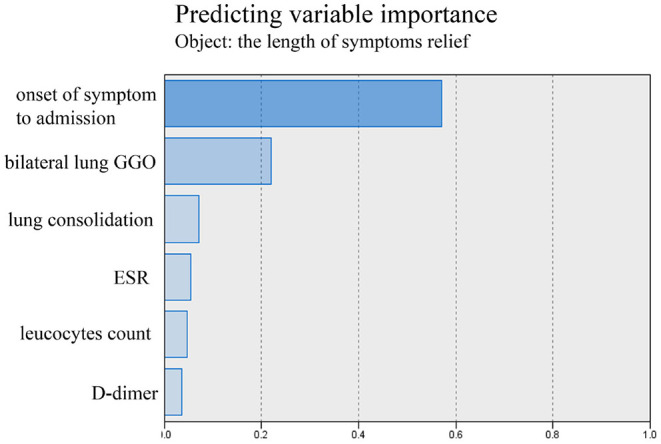
Using automatic linear modeling to predict relevant factors of the length of symptoms relief.

The tests of inflammation (leucocytes count, C-reactive protein, ESR), systemic damage (LDH, CK), lymphopenia (lymphocytes count), coagulopathy (D-dimer), and lung injuries (bilateral lung GGO and lung consolidation) were reported to be related to the severity of COVID-19 (17–20). In order to value the length of hospitalization and symptoms relief, we analyzed these above clinical indicators together with onset of symptoms to admission and demography (gender and age). The length of hospitalization and symptoms relief were similar to normal distributions (Figures 1, 2), so we used automatic linear modeling to predict the relevant factors. We found that factors related to the length of hospitalization included onset of symptoms to admission, ESR, CPR, leucocytes count, bilateral lung GGO, and lung consolidation (Figure 3); factors related to the length of symptoms relief included onset of symptoms to admission, ESR, D-dimer, leucocytes count, bilateral lung GGO, and lung consolidation (Figure 4). Then multiple linear regression analysis was used to confirm the results. As to the length of hospitalization, the R value and R square value were 0.658 and 0.433, respectively (Table 5). As to the length of symptoms relief, the R value and R square value were 0.680 and 0.462, respectively (Table 6). The results indicated that these relevant factors were linearly related to the length of hospitalization and symptoms relief, and can explain more than 40% of the variability of them. But the results of regression coefficient (β) showed that onset of symptoms to admission, ESR, leucocytes count, and bilateral lung GGO were linearly related to the length of hospitalization (*P* < 0.05); onset of symptoms to admission, leucocytes count, bilateral lung GGO, and lung consolidation were linearly related to the length of symptoms relief (*P* < 0.05) (Tables 5, 6). Except for bilateral lung GGO, onset of symptoms to admission, ESR, and leucocytes count were negatively correlated with the length of hospitalization (Tables 5, 6). And except for leucocytes count, onset of symptoms to admission, bilateral lung GGO, and lung consolidation were positively correlated with the length of symptoms relief (Tables 5, 6).

**Table 5:** Relevant factors of the length of hospitalization.

***R*-value**	**R square value**	**Adjusted R square value**	**Parameters**	**β value**	***P*-value**
0.658	0.433	0.399	Onset of symptoms to admission	−0.273	0.044
			Bilateral lung GGO	7.154	0.000
			ESR	−0.063	0.024
			Leucocytes count	−0.613	0.024
			Pulmonary consolidation	2.724	0.071
			CRP	0.758	0.086

**Table 6:** Relevant factors of the length of symptoms relief.

***R*-value**	**R square value**	**Adjusted R square value**	**Parameters**	**β value**	***P*-value**
0.680	0.462	0.430	Onset of symptoms to admission	0.708	0.000
			Bilateral lung GGO	7.417	0.000
			ESR	−0.052	0.052
			Leucocytes count	−0.585	0.031
			Pulmonary consolidation	3.783	0.008
			D-dimer	0.447	0.217

### Analyzing the Difference of the Length of Hospitalization and Symptoms Relief of Inpatients With Moderate-Type COVID-19 in Scoring Groups

In order to conveniently use these related factors for clinical evaluation, we defined that when the regression coefficient (β) was <0.05, the related factor scored 1 point; when the regression coefficient (β) was <0.01, the related factor scored 2 points. Combining the clinical situation and the results of multiple linear regression analysis, the scoring criteria of relevant factors for the length of hospitalization and symptoms relief are described in Table 7, and all other conditions scored 0 points. The total correlation score was calculated for each patient by summation of the score points (Supplementary Materials). Then comparisons between the total score groups were analyzed with one-way ANOVA. With increasing scores, the time of hospitalization and symptoms relief of inpatients with moderate-type COVID-19 lengthened (Tables 8, 9). Compared with the 0 and 1 scoring groups, other scoring groups had a longer time of hospitalization (*P* < 0.01); except the 5 scoring group, the length of hospitalization in the 4 scoring group was longer than other scoring groups (*P* < 0.01) (Table 10). Compared with the 0 scoring group, other scoring groups had a longer time of symptoms relief, except the 1 scoring group (*P* < 0.01). Compared with the 1 and 2 scoring groups, the length of symptoms relief in above 4 scoring groups was longer (*P* < 0.1). Compared with the 3 and 4 scoring groups, the length of symptoms relief in the 6 scoring group was longer (*P* < 0.05) (Table 11).

**Table 7:** The scoring criteria for the length of hospitalization and symptoms relief.

	**Parameters**	**Scoring**		**Parameters**	**Scoring**
The length of hospitalization	Onset of symptoms to admission	1	The length of symptoms relief	Onset of symptoms to admission	2
	<6 (days)			≥6 (days)	
	Bilateral lung GGO	2		Bilateral lung GGO	2
	Normal ESR (0~15mm/h)	1		Pulmonary consolidation	2
	Reduced leucocytes count (<3.5×10^9^/L)	1		Reduced leucocytes count (<3.5×10^9^/L)	1

*Remarks: The average of onset of symptoms to admission in the 107 patients was 6 days*.

**Table 8:** The length of hospitalization in the scoring groups.

	**The total correlation scores**	**Patients**	**The length of hospitalization (mean ± SEM)**
The length of hospitalization	0	15	10 ± 4
	1	17	13 ± 7
	2	26	19 ± 7
	3	24	19 ± 6
	4	22	24 ± 6
	5	3	24 ± 7

**Table 9:** The length of symptoms relief in the scoring groups.

	**The total correlation scores**	**Patients**	**The length of symptoms relief (mean ± SEM)**
The length of symptoms relief	0	18	17 ± 6
	1	6	20 ± 8
	2	42	22 ± 6
	3	9	27 ± 7
	4	15	26 ± 7
	5	3	31 ± 12
	6	10	33 ± 7
	7	4	29 ± 4

**Table 10:** The comparison of scoring groups over the length of hospitalization.

	**The length of hospitalization (*P*-value)**		**The length of hospitalization (*P*-value)**
0 scoring group VS 1 scoring group	0.212	1 scoring group VS 5 scoring group	0.010
0 scoring group VS 2 scoring group	0.000	2 scoring group VS 3 scoring group	0.887
0 scoring group VS 3 scoring group	0.000	2 scoring group VS 4 scoring group	0.004
0 scoring group VS 4 scoring group	0.000	2 scoring group VS 5 scoring group	0.201
0 scoring group VS 5 scoring group	0.001	3 scoring group VS 4 scoring group	0.007
1 scoring group VS 2 scoring group	0.007	3 scoring group VS 5 scoring group	0.227
1 scoring group VS 3 scoring group	0.005	4 scoring group VS 5 scoring group	0.915
1 scoring group VS 4 scoring group	0.000		

**Table 11:** The comparison of scoring groups over the length of symptoms relief.

	**The length of symptoms relief (*P*-value)**		**The length of symptoms relief (*P*-value)**
0 scoring group VS 1 scoring group	0.288	2 scoring group VS 4 scoring group	0.048
0 scoring group VS 2 scoring group	0.005	2 scoring group VS 5 scoring group	0.040
0 scoring group VS 3 scoring group	0.000	2 scoring group VS 6 scoring group	0.000
0 scoring group VS 4 scoring group	0.000	2 scoring group VS 7 scoring group	0.060
0 scoring group VS 5 scoring group	0.001	3 scoring group VS 4 scoring group	0.876
0 scoring group VS 6 scoring group	0.000	3 scoring group VS 5 scoring group	0.388
0 scoring group VS 7 scoring group	0.001	3 scoring group VS 6 scoring group	0.035
1 scoring group VS 2 scoring group	0.472	3 scoring group VS 7 scoring group	0.584
1 scoring group VS 3 scoring group	0.065	4 scoring group VS 5 scoring group	0.311
1 scoring group VS 4 scoring group	0.061	4 scoring group VS 6 scoring group	0.012
1 scoring group VS 5 scoring group	0.030	4 scoring group VS 7 scoring group	0.483
1 scoring group VS 6 scoring group	0.000	5 scoring group VS 6 scoring group	0.539
1 scoring group VS 7 scoring group	0.045	5 scoring group VS 7 scoring group	0.747
2 scoring group VS 3 scoring group	0.072	6 scoring group VS 7 scoring group	0.272

## Discussion

Pneumonia (COVID-19) caused by the infection of the new coronavirus (SARS-CoV-2) has become the first worldwide pandemic disease in the 21st century (21). The genomic characterization suggested that SARS-CoV-2 belonged to the beta coronavirus family (22). However, it is more contagious than SARS-COV (6), and has infected about 119,000,000 people around the world up to now, posting a serious burden on the world health system. In order to quickly cut off the spread of the virus, effectively treating infected patients is the key to control the outbreak of COVID-19. Our study and other studies showed that a large number of patients had moderate-type COVID-19 (11–15). However, the relevant factors for disease progression in patients with moderate-type COVID-19 were not completely clear. This study described the characteristics of patients with moderate-type COVID-19 in the outbreak area (Wuhan) and the imported area (Yiyang), and found that onset of symptoms to admission, ESR, leucocytes count, and bilateral lung GGO were linearly related to the length of hospitalization; onset of symptoms to admission, leucocytes count, bilateral lung GGO, and lung consolidation were linearly related to the length of symptoms relief. We also offered an effective scoring system to predict the length of hospitalization and symptoms relief, which could help to determine the severity and treatment of moderate-type patients with COVID-19.

Our study enrolled 71 ordinary patients (28 inpatients from Wuhan and 43 inpatients from Yiyang) with moderate-type COVID-19, and about 20% of them contracted or previously had comorbidities. However, a previous report from Wuhan showed a larger number of COVID-19 patients (30%) with comorbidities (23). The possible reason was that severe patients were excluded in this study. D-dimer [Wuhan 9 (32.14%) vs. Yiyang 12 (27.9%)], C-reactive protein [Wuhan 15 (53.57%) vs. Yiyang 12 (27.9%)], and ESR [Wuhan 20 (71.43%) vs. Yiyang 30 (69.8%)] were increased in more than 20% of the ordinary patients. Lymphopenia occurred in 22 ordinary patients [78.57%] from Wuhan and 16 [37.2%] from Yiyang. These results suggested that ordinary patients with moderate-type COVID-19 may develop abnormal coagulation, inflammation, and immunodeficiency. Results of chest CT scans indicated that bilateral lung GGO and lung consolidation were observed more in the ordinary patients with moderate-type COVID-19 from Wuhan than in those from Yiyang, suggesting that the ordinary patients in Wuhan had more severe lung injuries. From the results of serum tests and CT scans, we found that the ordinary patients with moderate-type COVID-19 in Wuhan (the outbreak area) had more serious disease than those patients in Yiyang (the imported area). The reasons may be as follows: Wuhan was the outbreak area; the virus loads invading the patient were greater; and the chance of repeated infection of patients was increased. The virus loads are related to the severity of COVID-19, which may contribute to the guidance on clinical diagnosis and treatment (24). However, there were no relevant studies to clearly explain this phenomenon. Except antiviral treatment, ordinary patients from Yiyang received fewer other treatments than those from Wuhan, suggesting that the treatment between the outbreak area and the imported area was different. Although the treatment of ordinary inpatients in Wuhan had been strengthened, the length of hospitalization and symptoms relief were still much longer, which implied systemic glucocorticoids and intravenous immunoglobulin cannot effectively clear the SARS-CoV-2 infection and shorten the course of disease.

In addition, we compared the clinical course of ordinary patients (28 cases) and infected medical staff (36 cases) from Wuhan. When displaying symptoms, medical staff would go to hospital more promptly than ordinary patients [6 (IQR 3.25–8.75] vs. 3 (IQR 2–5.75)], which may be due to occupational factors. Similar to ordinary patients, the most common symptoms of infected medical staff were fever and cough. However, the early symptoms of viral infection (fatigue and myalgia) were more likely to appear in infected medical staff, due to their earlier hospitalization. Compared with infected medical staff, the indicators of organ damage (AST, LDH) and inflammation (C-reactive protein, ESR) in ordinary patients were significantly increased, and lymphocytes were significantly reduced. Furthermore, chest CT scans showed that ordinary patients had more bilateral lung GGO and lung consolidation. These results of serum tests and imaging suggested that the ordinary patients in Wuhan were more serious ill than infected medical staff. Part of the reason may be that infected medical staff were younger and had no underlying diseases, which were reported to be related to the severity of COVID-19 (9). The occupational factors also allowed infected medical staff to protect themselves better and seek medical treatment quickly, which implied that protective measures and timely medical treatment might inhibit the progress of COVID-19 and reduce the severity of the disease. Consistent with the severity of COVID-19, the length of hospitalization and symptoms relief were longer in ordinary patients with moderate-type COVID-19.

According to the above results, compared to infected medical staff from Wuhan and ordinary inpatients with moderate-type COVID-19 from Yiyang, lymphopenia, elevated C-reactive protein, increased LDH, bilateral lung GGO, and lung consolidation were more likely to appear in ordinary inpatients with moderate-type COVID-19 from Wuhan. These indicators were reported to be related to the severity of the pneumonia (25–27). So the length of hospitalization and symptoms relief were longer in ordinary patients with moderate-type COVID-19 from Wuhan.

In this study, apart from describing the characteristics of patients with moderate-type COVID-19 in the outbreak area (Wuhan) and the imported area (Yiyang), we also found that onset of symptoms to admission, ESR, leucocytes count, and bilateral lung GGO were linearly related to the length of hospitalization; onset of symptoms to admission, leucocytes count, bilateral lung GGO, and lung consolidation were linearly related to the length of symptoms relief. Onset of symptoms to admission was negatively correlated with the length of hospitalization and positively correlated with the length of symptoms relief. The result suggested that some moderate-type patients may recover without treatment, which was consistent with the US CDC COVID-19 guidelines (16). But prompt medical treatment can shorten the process of this disease. Normal ESR and reduced leucocytes count seemed to accompany a longer length of hospitalization, and leucocytes count was also negatively correlated with the length of symptoms relief. Leucocytes count was an important indicator related to inflammation and immunity (28, 29), so we speculated that appropriate inflammation and immunity may help moderate patients clear the virus. Ali A Ghweil and his colleagues reported that reduced leucocytes count was more likely to appear in patients with severe COVID-19 (30). Functional exhaustion of both innate and adaptive immune response was positively related to the severity of COVID-19 (31). The World Health Organization (WHO) has published guidance recommending no corticosteroids for those with non-severe COVID-19 (32), which seemed to prove our speculation to some extent. Different from our results, ESR was reported to be positively related to the severity of this disease in some studies (33–35). The reason might be that severe-type patients with COVID-19 were also included in these studies. Lung injuries (bilateral lung GGO or lung consolidation) have been confirmed to be an important indicator of the severity of this disease (27), as they were accompanied with a longer period of hospitalization and symptoms relief. Moreover, the total correlation scores can work well to evaluate the length of hospitalization and symptoms relief in all 107 inpatients with moderate-type COVID-19. With increasing scores, the time of hospitalization and symptoms relief of inpatients lengthened. The results suggested that the scoring system was an effective instrument to determine the severity and the treatment of COVID-19.

## Limitations

There were several limitations in this study. First, the study did not include patients with moderate-type COVID-19 who worsened or died. Second, since the patients in this study were not severe, the attending physicians reviewed the serum tests and imaging examinations less frequently and not in full during the hospitalization. So we could not describe the changes of the treatment process of these patients in detail. Third, due to the small number of cases in this study, multicenter or nationwide studies are warranted to validate these findings.

## Conclusions

This study described the clinical course of patients with moderate-type COVID-19, and found that patients with moderate-type COVID-19 in outbreak areas had a more serious infection and needed stronger treatment and longer treatment time. Onset of symptoms to admission, ESR, leucocytes count, and bilateral lung GGO can be effective predictors of the length of hospitalization. And onset of symptoms to admission, leucocytes count, bilateral lung GGO, and lung consolidation can be effective predictors of the length of symptoms relief. Most importantly, we have created an effective scoring system to determine the length of hospitalization and symptoms relief in inpatients with moderate-type COVID-19, which could contribute to the diagnosis and treatment of COVID-19.

## Data Availability Statement

The original contributions presented in the study are included in the article/Supplementary Material, further inquiries can be directed to the corresponding authors.

## Ethics Statement

The studies involving human participants were reviewed and approved by the Ethic Committee of Central Hospital of Wuhan; the Ethic Committee of Fourth People's Hospital. The patients/participants provided their written informed consent to participate in this study.

## Author Contributions

XLi, XLv, CS, and JM collected the epidemiological and clinical data. XLi, XLv, YZ, and YJ summarized all data. XLi, MJ, RH, and ML drafted the manuscript. JM and XLi revised the final manuscript. All authors contributed to the article and approved the submitted version.

## Conflict of Interest

The authors declare that the research was conducted in the absence of any commercial or financial relationships that could be construed as a potential conflict of interest.
