# Supplementary material for: A Retrospective Cohort Study on the Clinical Course of Patients With Moderate-Type COVID-19
Source: Front Public Health. 2021 Apr 26;9:593109. doi: 10.3389/fpubh.2021.593109 (PMC8112071; doi:10.3389/fpubh.2021.593109)
Supplement: Supplementary file 1 [file Data_Sheet_1.doc]

Supplementary table1：Demographic, clinical characteristics, treatment and outcome of ordinary patients with moderate type COVID-19 from Yiyang

|  | **16 patients with Wuhan**  **travel history** | **27 patients without Wuhan**  **travel history** | **P value** |
| --- | --- | --- | --- |
| **Onset of symptom to admission,median(IQR), d** | 7(3.75-11), range from 2 to 21 | 3(2-7), range from 0 to 29 | 0.037 |
| **Age,median(IQR),years** | 46(33.25-54.25),range from 21 to 58 | 49(39-57),range from 15 to 80 | 0.428 |
| **Sex** |  |  |  |
| **male** | 10(62.5%) | 7(25.93%) | 0.018 |
| **female** | 6(37.5%) | 20(74.07%) |  |
| **Sgins and symtoms** |  |  |  |
| **Fever** | 5(31.25%) | 10(37.04%) | 0.700 |
| **Cough** | 10(62.5%) | 15(55.56%) | 0.655 |
| **Sputum** | 4(25%) | 9(33.33%) | 0.817 |
| **Dyspnea** | 0 | 1(3.70%) | 1 |
| **Fatigue** | 0 | 10(37.04%) | 0.016 |
| **Myalgia** | 2(12.5%) | 3(11.11%) | 1 |
| **Headache** | 2(12.5%) | 1(3.70%) | 0.635 |
| **Diarrhea** | 2(12.5%) | 0 | 0.133 |
| **Dizziness** | 1(6.25%) | 3(11.11%) | 1 |
| **No symptoms** | 3(18.75%) | 4(14.81%) | 1 |
| **Comorbidities** |  |  |  |
| **Coronary atherosclerosis** | 0 | 3(11.11%) | 0.445 |
| **Hypertension** | 1(6.25%) | 3(11.11%) | 1 |
| **Diabetes** | 0 | 3(11.11%) | 0.445 |
| **Treatment** |  |  |  |
| **normal flow of oxygen** | 0 | 3(11.11%) | 0.445 |
| **Glucocorticoids** | 2(12.5%) | 7(25.93%) | 0.510 |
| **Intravenous immunoglobulin therapy** | 1(6.25%) | 4(14.81%) | 0.723 |
| **Antibiotic treatment** | 6(37.5%) | 11(40.74%) | 0.834 |
| **Antiviral treatment** | 16(100%) | 27(100%) |  |
| **Clinical outcome** |  |  |  |
| **Discharged** | 16(100%) | 27(100%) |  |
| **The length of hospitalization (IQR),d** | 12(8.25-15), range from 4 to 19 | 10(7-15), range from 4 to 20 | 0.640 |
| **The length of symptoms relief (IQR),d** | 18(15-21.5), range from 12 to 39 | 15(11-19), range from 9 to 38 | 0.057 |

Supplementary table2: Laboratory examination of ordinary patients with moderate type COVID-19 from Yiyang

|  | **16 patients with Wuhan**  **travel history** | **27 patients without Wuhan**  **travel history** | **P value** |
| --- | --- | --- | --- |
| **Leucocytes count(×10^9^/L; normal range 3.5~9.5)(IQR)** | 5.36(4.05-7.14) | 6.26(4.10-8.15) | 0.421 |
| **Increased** | 1(6.25%) | 3(11.11%) | 1 |
| **Lymphocytes count(×10^9^/L; normal range 1.1~3.2)(IQR)** | 1.27(1.02-1.47) | 1.16(0.84-1.67) | 0.393 |
| **Decreased** | 4(25%) | 12(44.44%) | 0.202 |
| **Neutrophil count(×10^9^/L; normal range 1.8~6.3)(IQR)** | 3.04(2.24-5.29) | 4.19(2.26-5.59) | 0.327 |
| **Increased** | 1(6.25%) | 4(14.81%) | 0.723 |
| **Decreased** | 1(6.25%) | 2(7.41%) | 1 |
| **Lymphocytes count/Neutrophil count(IQR)** | 0.39(0.32-0.57) | 0.29(0.17-0.55) | 0.148 |
| **Monocyte count(×10^9^/L; normal range 0.1~0.6)(IQR)** | 0.42(0.33-0.56) | 0.47(0.30-0.57) | 0.890 |
| **Increased** | 3(18.75%) | 3(11.11%) | 0.808 |
| **Platelet count(×10^9^/L; normal range 125~350)(IQR)** | 196(156-223) | 170(145-228) | 0.416 |
| **Decreased** | 2(12.5%) | 3(11.11%) | 1 |
| **Hemoglobin(g/L; normal range 130~175)(IQR)** | 127(116-134) | 123(112-134) | 0.684 |
| **Decreased** | 9(56.25%) | 18(66.67%) | 0.495 |
| **D-dimer(mg/L;normal range 0~0.5)(IQR)** | 0.41(0.16-0.75) | 0.37(0.15-0.68) | 0.870 |
| **Increased** | 5(31.25%) | 7(25.93%) | 0.980 |
| **Aspartate aminotransferase(U/L;normal range 15~40)(IQR)** | 25(14.75-34.25) | 24(20-34) | 0.451 |
| **Increased** | 1(6.25%) | 3(11.11%) | 1 |
| **Blood urea nitrogen(mmol/L;normal range 3.6~9.5)(IQR)** | 4.16(3.20-4.90) | 3.83(3.26-5.27) | 0.940 |
| **Increased** | 0 | 1(3.70%) | 1 |
| **Decreased** | 7(43.75%) | 7(25.93%) | 0.228 |
| **Blood creatinine(umol/L;normal range 64~104)(IQR)** | 68.70(56.93-82.45) | 65.1(52.5-74.8) | 0.466 |
| **Increased** | 0 | 1(3.70%) | 1 |
| **Decreased** | 7(43.75%) | 13(48.15%) | 0.780 |
| **C-reactive protein(mg/dL;normal range 0~0.6)(IQR)** | 0.12(0.05-0.78) | 0.15(0.05-1.20) | 0.772 |
| **Increased** | 4(25%) | 8(29.63%) | 1 |
| **Erythrocyte sedimentation rate (mm/h; normal range 0~15)(IQR)** | 25.75(13.05-49.37) | 22(12-41.7) | 0.706 |
| **Increased** | 10(62.5%) | 20(74.07%) | 0.649 |
| **Creatine kinase(U/L;normal range 0~171)(IQR)** | 79.00(15.93-187.75) | 53(43.75-73.25) | 0.543 |
| **Increased** | 4(25%) | 1(3.70%) | 0.107 |
| **Lactate dehydrogenase(U/L;normal range 120~250)(IQR)** | 181.5(164-225) | 180(154-215) | 0.624 |
| **Increased** | 2(12.5%) | 1(3.70%) | 0.635 |
| **Chest CT scans** |  |  |  |
| **Bilateral lung GGO** | 2(12.5%) | 8(29.63%) | 0.362 |
| **Single lung GGO** | 7(43.74%) | 7(25.93%) | 0.228 |
| **Pulmonary consolidation** | 3(18.75%) | 3(11.11%) | p=0.808 |
